# Two-Dimensional Metallic NiSe_2_ Nanoclusters–Based Low-Cost, Flexible, Amperometric Sensor for Detection of Neurological Drug Carbamazepine in Human Sweat Samples

**DOI:** 10.3389/fchem.2020.00337

**Published:** 2020-04-30

**Authors:** Sushmitha Veeralingam, Sushmee Badhulika

**Affiliations:** Department of Electrical Engineering, Indian Institute of Technology Hyderabad, Hyderabad, India

**Keywords:** TMDCs, 2D metallic NiSe_2_, electrochemical sensor, flexible sensor device, carbamazepine

## Abstract

Here we report a low-cost, flexible amperometric sensing platform for highly selective and sensitive detection of carbamazepine (CBZ) in human sweat samples. Detailed morphological characterization of the two-dimensional transition metal dichalcogenide NiSe_2_, synthesized using one-step hydrothermal method, confirms the formation of dense NiSe_2_ nanoclusters in the range of 500–650 nm, whereas X-ray diffraction and X-ray photoelectron spectroscopy studies reveal a stable and pure cubic crystalline phase of NiSe_2_. The sensor device is fabricated by uniformly depositing an optimized weight percentage of as-synthesized NiSe_2_ onto flexible and biocompatible polyimide substrate using spin coating, and metal contacts are established using thermal evaporation technique. The sensor exhibits a remarkable sensitivity of 65.65 μA/nM over a wide linear range of 50 nM to 10 μM CBZ concentrations and a low limit of detection of 18.2 nM. The sensing mechanism and excellent response of NiSe_2_ toward CBZ can be attributed to the highly conductive metallic NiSe_2_, large electroactive surface area of its nanoclusters, and highly interactive Ni^2+^/Ni^3+^ oxidation states. Furthermore, the presence of 10-fold excess of capable interferents, such as lactic acid, glucose, uric acid, and ascorbic acid, does not affect the accurate determination of CBZ, thus demonstrating excellent selectivity. The real-time detection of CBZ is evaluated in human sweat samples using standard addition method, which yields reliable results. Furthermore, the sensor shows excellent robustness when subject to bending cycles and fast response time of 2 s. The strategy outlined here is useful in developing sensing platforms at low potential without the use of enzymes or redox binders for applications in healthcare.

## Introduction

Carbamazepine (CBZ) is a widely used therapeutic neurological drug prescribed for psychomotor seizures, clonic, and partial seizures, trigeminal neuralgia, and so on. Recent reports on antiepileptic drugs discuss dramatic side effects of CBZ including hyponatremia, bone alterations, and renal and pulmonary abnormalities leading to liver failure (Brueck et al., 2019; Jacobs et al., 2019; Nicoletti et al., 2019). Therapeutic intake of CBZ concentrations in excess of 0.5 μg/mL results in human deaths due to dissemination of antibiotic resistance genes (Wang et al., 2019). Carbamazepine is also reported to spread antiresistance genes in various biofluids, such as sweat, blood, and so on, which resist antimicrobial agents, thus causing microbe-related infections. Hence, monitoring of CBZ is crucial in healthcare and requires a user-friendly flexible platform to detect the concentrations of CBZ in human sweat.

Several analytical techniques have been developed for CBZ sensing including fluorescence probing (Ma et al., 2018), liquid chromatography (Yan and Row, 2006), and electrochemical methods such as cyclic voltammetry (Veiga et al., 2010), differential pulse voltammetry (DPV) (Lin et al., 2012), amperometric techniques (De Carlo et al., 2015), enzyme modified immunoassay, and so on (Contin et al., 1985). However, most of these analytical techniques involve complicated sample preparation steps, resulting in unspecified impurities, and the sample disregard limit is high, thus resulting in CBZ waste generation (Trišović et al., 2014) in the test solutions. Further, measurement of the enzyme activity is complex; biorecognition of the analyte is crucial, and sensitivity was found to be very less in enzyme-modified immunoassays (Paxton, 1982). Among electrochemical techniques, amperometric approach is preferable because of several advantages, such as (i) reduced sample, (ii) quicker response time, and (iii) the possibility of obtaining high sensitivity with easily reducible or oxidizable species and so on (Wang, 1999).

Two-dimensional (2D) transition metal dichalcogenides (TMDCs), such as WS_2_, SnSe_2_, FeS_2_, and TaSe_2_, have emerged as potential replacements for organic and silicon-based materials owing to their excellent physical, chemical, and electron transport properties (Ma et al., 2017; Veeralingam et al., 2019a,b,c, 2020; Veeralingam and Badhulika, 2020a). NiSe_2_, a layered metallic TMDC, possesses unique physical, optical, and electrochemical properties viz., high carrier mobility, and large surface-to-volume ratio that makes it well-suited for applications such as batteries and supercapacitors (Wang et al., 2017). In particular, the zero bandgap and intrinsic electrical conductivity (Swesi et al., 2017) of NiSe_2_ structure make it a potential candidate for excellent electron transfer–assisted sensing applications. NiSe_2_ has been synthesized using various methods such as direct stoichiometric process (Anantharaj et al., 2019), precipitation (Mani et al., 2017), thermal decomposition, and hydrothermal (Veeralingam et al., 2019a), and solvothermal reactions (Yu et al., 2017). However, precipitation technique requires intermittent processing steps due to slow diffusion and insolubility of Se atoms. The thermal decomposition leads to the formation of undesired Ni element as impurity. Moreover, the stoichiometric elemental composition and external temperature play a crucial role in determining the phase of the 2D-NiSe_2_. In contrast, hydrothermal synthesis is a simple, low-cost, facile synthesis technique with excellent dispersion of precursors in aqueous medium assisting the formation of pure crystalline nanostructures. Furthermore, layered 2D-NiSe_2_ can be easily drawn into desired morphologies by designing unique hierarchical architectures using flexible substrates (Vishnu et al., 2019). Thus, it would be interesting to explore NiSe_2_ on flexible/biocompatible substrates for electrochemical sensing of CBZ using hydrothermal synthesis.

In this work, a one-step hydrothermal method is employed to synthesize layered metallic NiSe_2_ nanoclusters for highly sensitive detection of CBZ in human sweat samples. NiSe_2_ was uniformly drop-casted on polyimide substrate for fabricating a flexible platform for conformal human skin sensors. The NiSe_2_ nanoclusters with inherently large surface area displayed remarkable sensitivity toward a wide linear range of CBZ concentrations from 50 nM to 1 μM. The CBZ sensing was performed using amperometry technique. The interference studies displayed excellent selectivity of NiSe_2_ nanoclusters toward CBZ against other interfering co-analytes, and the real-time detection CBZ is evaluated in human sweat samples employing standard addition (SA) method. Furthermore, the sensor shows excellent robustness when subject to bending cycles and fast response time of 2 s. The strategy employed here displays enhanced performance of the NiSe_2_ sensor surpassing the previously reported CBZ sensors, thus paving ways for development of low-cost, flexible sensors for point-of-care diagnostic devices.

## Experimental

### Materials

Dimethyl formaldehyde (DMF), selenium powder (Se), and nickel chloride (NiCl_2_), sodium borohydride (NaBH_4_), polyimide tape, NaCl, KCl, Na_2_HPO_4_, KH_2_PO_4_, and 99.99% pure copper metal were procured from Sigma-Aldrich (India) and were used as received. Deionised (DI) water was collected from Millipore system.

### Instrumentation

X-ray photoelectron spectroscopy (XPS) studies were carried out using ULVAC–PHI 5000, Versa Probe II (ULVAC-PHI Inc. - Japan). Scanning electron microscopy (SEM) analysis was performed by Carl Zeiss Ultra-55 SEM (FELMI-JFE - Germany). Structural studies were done using X'pert PRO X-ray diffraction (XRD) (PANalytical Products - India) with Cu Kα radiation. Differential pulse voltammetry studies were performed using CHI 660E electrochemical workstation (CH Instruments India) at room temperature using a three-electrode cell setup consisting of NiSe_2_ on glassy carbon electrode (GCE) as the working electrode, 1 M KCl Ag|AgCl as the reference electrode, and a platinum wire electrode as the counter electrode. All electrical measurements were performed using Keithley 2450 SMU instrument (Tektronics - India).

### Synthesis and Device Fabrication

#### Synthesis of NiSe_2_ Nanoclusters

NiSe_2_ nanoclusters were synthesized using facile one-step hydrothermal technique. Briefly, 0.09 M of Se and 0.1 M of NaBH_4_ were added to DI water and stirred for 3 h after a black dispersed solution is obtained. Further, 0.08 M of NiCl_2_ was added to the Se solution and stirred for 1 h until completely dissolved. Thereafter, the solution was transferred to a 50 mL Teflon lined autoclave and was maintained at 180°C for 24 h. The reactor was allowed to cool down, and the resultant NiSe_2_ was centrifuged and dried overnight at 70°C. The obtained NiSe_2_ powder was collected and used for device fabrication.

#### Fabrication of Flexible NiSe_2_/Polyimide Device

The as-synthesized NiSe_2_ nanoparticles were uniformly dispersed in DMF solution and deposited on the cleaned polyimide substrate (1 × 1 cm) using spin coating technique. The optimized weight % of 0.2 of NiSe_2_ was dispersed in DMF and coated at 500 rotations per minute (rpm) for 1 min to deposit a seeding layer on the polyimide (PI) substrate. Details of weight percentage–based optimization details can be found in Supplementary Information Section S1. The seed-coated device was dried at 70°C for 3 h. Further, the NiSe_2_ thin film was deposited at 2,000 rpm and calcined at 70°C for 3 h. The spin-coated NiSe_2_ films were masked in the center measuring 0.5 × 0.5 cm, and the copper contacts were evaporated on both sides (0.5 × 0.25 cm) as source and drain. For thermal evaporation, the vacuum was maintained at 3.5 × 10^−6^ bar and copper metal was thermally evaporated at an evaporation rate of 0.35 kÅ per minute for 30 s. The fabricated devices were used for CBZ sensing.

### Sample Preparation for CBZ Sensing

Phosphate-buffered solution (pH 7.2) was prepared by mixing 68.44 mM of NaCl, 0.134 mM of KCl, 5.07 mM of Na_2_HPO_4_, and 88.1 mM of KH_2_PO_4_ in 2 mL of DI water. Simulated sweat was prepared by adding 5 mg/mL NaCl, 4 mg/mL urea, and 1 mM of lactic acid (LA) with a final measured pH of 3.0 in deionized water. Various concentrations of CBZ were prepared in phosphate-buffered solution for amperometry studies and prepared in sweat solutions for real-time analysis.

## Results and Discussion

### Physiochemical Characterization of NiSe_2_

**Figure 1:**
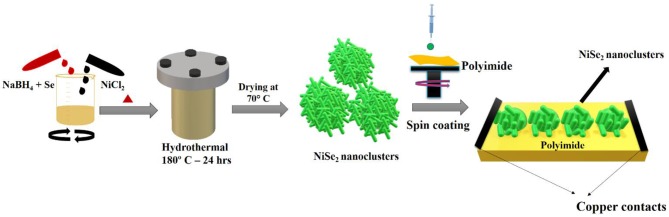
Schematic diagram for the hydrothermal synthesis of NiSe_2_ nanoclusters followed by fabrication of NiSe_2_/PI device.

**Figure 2:**
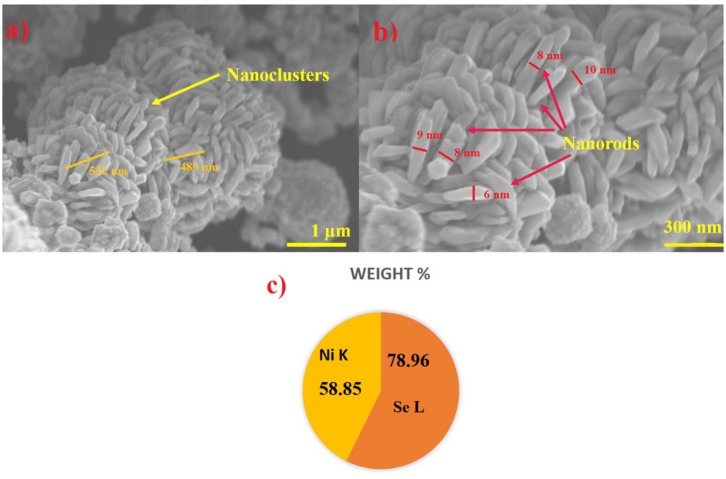
**(a)** Low-magnification SEM images of NiSe_2_ nanoclusters. **(b)** High-magnification SEM images displaying nanorod-like structure within the nanocluster. **(c)** Elemental composition of Ni and Se atoms in NiSe_2_.

The schematic illustrating the synthesis of NiSe_2_ nanoclusters and fabrication of NiSe_2_/PI device is shown in Figure 1. The morphology of hydrothermally synthesized NiSe_2_ nanoclusters was analyzed using SEM characterization. Figure 2a displays the low-magnification images of as-synthesized NiSe_2_ nanoclusters. The image displays the uniform distribution of nanoclusters-like structure. The average size of the nanoclusters was found to be in the range of 500–650 nm wherein nanorods were combined together to form nanoclusters. Figure 2b illustrates the high-magnification images of synthesized NiSe_2_ nanoclusters. Nanorods-like structures were clearly observed from the high-magnification images. The formation of hierarchical nanoclusters can be attributed to the anisotropic growth factor and surface energy. The surface energy of NiSe_2_ was reduced at a hydrothermal temperature of 180°C, assisting in the formation of aggregated nanoclusters. Moreover, it is evident from the SEM images that nanoclusters are composed of tiny nanorods. The nanorods-like morphology can be attributed to the crystal structure of NiSe_2_, which was later found to be cubic in nature corroborating well with the XRD studies. Figure 2c displays the elemental composition of NiSe_2_ nanoclusters. The wt. % of Ni and Se atoms was found to be 58.85 and 78.96%, respectively. This stoichiometric wt. % ratio was approximately in the range of 1:2 forming NiSe_2_ nanoclusters (Sobhani and Salavati-Niasari, 2014). This can be attributed to the optimum precursor concentration used in hydrothermal synthesis refluxed to form NiSe_2_.

**Figure 3:**
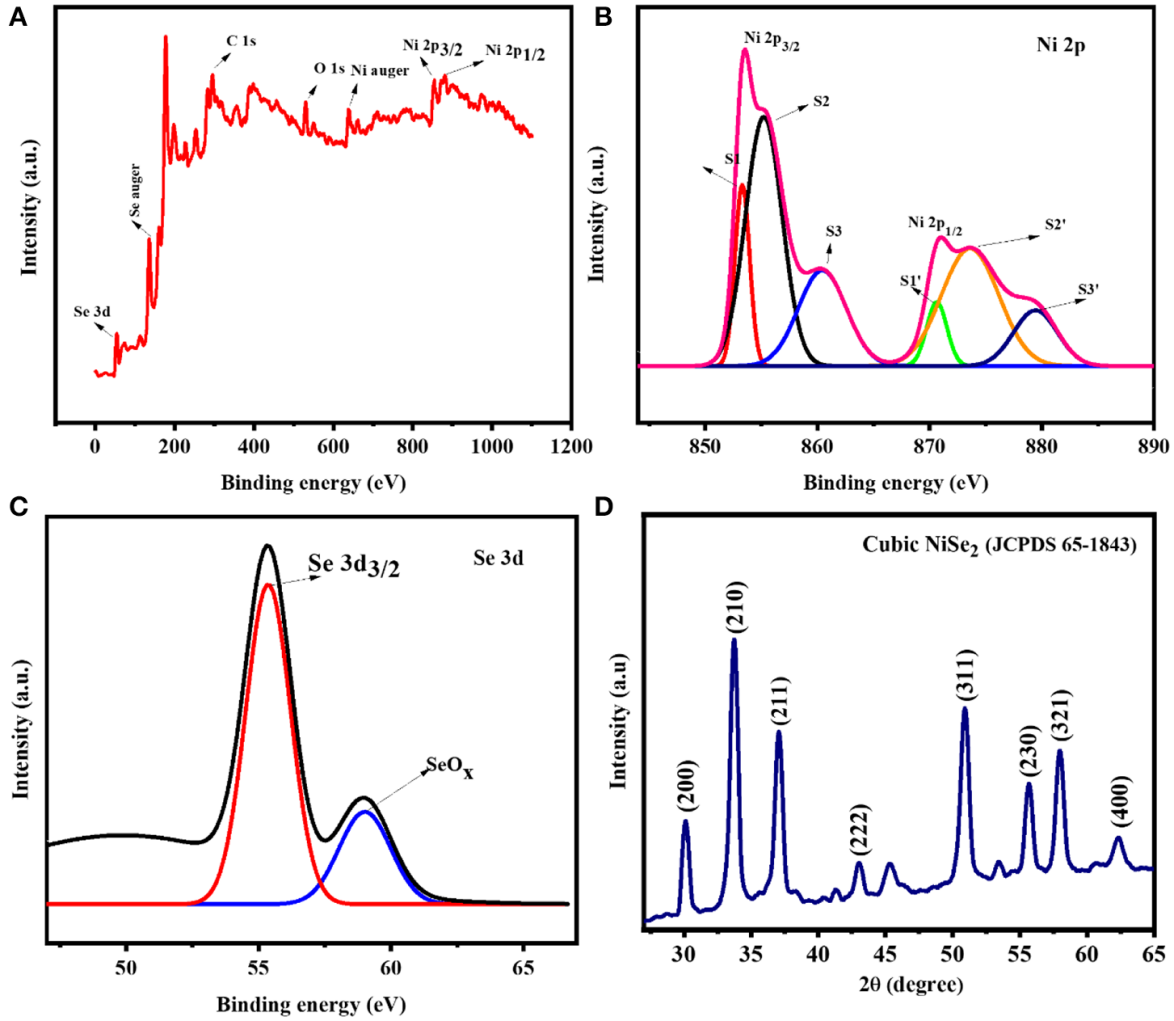
X-ray photoelectron spectroscopy spectrum of **(A)** complete spectrum of NiSe_2_, **(B)** deconvolution spectra of Ni 2p, **(C)** deconvolution spectrum of Se 3d, and **(D)** XRD patterns of the hierarchical NiSe_2_ nanoclusters.

In order to validate the surface sensitive quantitative chemical composition and the electronic states of the synthesized NiSe_2_, XPS studies were performed. Figure 3A displays the survey spectrum of four elements, namely, Ni 2p, Se 3d, C 1s, and O 1s. The presence of O 1s can be attributed to the surface oxidation states present on the NiSe_2_ surface. Figure 3B displays the deconvoluted spectra of Ni 2p with two main peaks corresponding to Ni 2p_3/2_ and Ni 2p_1/2_ at 852 and 870 eV, respectively. The major peaks can be further resolved into S1, S2, S3, and S1′, S2′, and S3′ peaks. The unreacted Ni^2+^ ions are represented by peaks at S1 (852.9 eV) and S1′ (870.2 eV). The peaks corresponding to S2 (854.8 eV) and S2′ (873.4 eV) represent Ni^3+^ ions, whereas the peaks at S3 (859.9 eV) and S3′ at (878 eV) validate the Ni^2+^ oxidation state. Figure 3C displays the deconvoluted spectra of Se 3d wherein the peak 55.7 eV displays the presence of Se${}_{2}^{2-}$ and peak at 59.1 eV is due to oxidation of Se atoms to SeO_x_. The binding energy obtained was used to evaluate the composition of Ni and Se. The approximate composition ratio was obtained as 1:2 validating the formation of NiSe_2_. The obtained XPS result corroborates well with the previous literature (Arul and Han, 2016). To understand the crystallographic orientations and lattice constant of the as-synthesized NiSe_2_ nanoclusters, XRD studies were performed. Figure 3D illustrates the XRD pattern of the synthesized NiSe_2_ nanoclusters. The diffraction peaks match well with the typical cubic NiSe_2_ structure with a lattice constant of *a* = 5.960 Å and with Pa$\overset{\bar{}}{3}$ point group. The diffraction peaks at 29°, 34°, 37°, 43°,50°, 56°, 57°, and 64° can be assigned to crystal planes of (200), (210), (211), (222), (311), (230), (321), and (400), respectively. The obtained XRD pattern can be indexed to JCPDS card no. 65-1843 (Zhang et al., 2019). The formation of NiSe_2_ is evident from the obtained XRD and XPS spectrum.

### Carbamazepine Sensing Based on I–V Studies

**Figure 4:**
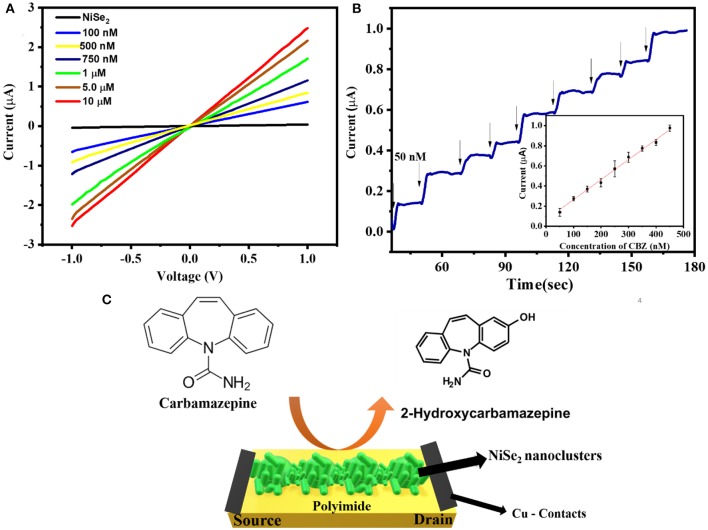
**(A)** The I–V characteristics of the NiSe_2_ device upon exposure to increase in concentrations of CBZ ranging from 100 nM to 10 μM. **(B)** Amperometric response of the fabricated NiSe_2_/PI sensor for successive increase in concentrations of 50 nM. **(C)** Schematics of the sensing mechanism of CBZ on the NiSe_2_/PI sensor.

Amperometric technique was employed for evaluating the response of NiSe_2_ sensor toward CBZ because it possesses several advantages such as high sensitivity, time-based response, and a quicker response time over other electrochemical techniques. Prior to that, the as-fabricated NiSe_2_/PI sensor was configured into the chemiresistive mode of sensing. Different concentrations of CBZ were drop-casted onto the NiSe_2_/PI sensor, and the corresponding current–voltage (I–V) characteristics were studied in the voltage range −1 to 1 V, as shown in Figure 4A. The device displayed a linear I–V characteristic, which indicated the ohmic electrical contacts between the copper contacts and NiSe_2_ device. Further, ln(I) vs. (V)^1/2^ was also plotted for the NiSe_2_/PI device as displayed in Figure S4. The results validate the ohmic type of behavior of NiSe_2_. This can be attributed to the barrier-free transfer of charge carriers in 2D-layered NiSe_2_ device, and due to the metallic nature of NiSe_2_, an enhanced electron conductivity was noted. Further, the prepared (explained in *Instrumentation*) different concentrations of CBZ solution were spiked on the device, and corresponding I–V measurements were taken. The CBZ concentrations ranging from 100 nM to 10 μM were spiked successively onto the device, and the response was collected from the device after 5 min, to obtain stabilized response. As a result, an increase in current was obtained for increase in concentrations of CBZ. The increase in current can be attributed to the oxidation of CBZ to 2-hydroxy CBZ (Jin et al., 2019) as shown in Figure 4C. This clearly demonstrates that metallic NiSe_2_ acts as an efficient electron mediator performing the function of an oxidizing agent. The initial step in oxidation of CBZ involves one electron oxidation of nitrogen atom to form a radical cation. This radical happens to be in a number of resonance forms (Chen et al., 2019). The electron gets transferred to NiSe_2_ from CBZ being responsible for increase in current flow through the device. Further, the SeO_x_ present on the surface also assists in the oxidization of CBZ molecules. The adsorbed oxygen atoms on the NiSe_2_ surface tend to react with Se atoms to form SeOx. Thus, oxygen atoms present in the surface reduces assisting in the enhancement of the active surface area available for interaction of CBZ molecules. The change in surface states due to the reacted oxygen atoms increases the carrier concentration on the surface of NiSe_2_, which further increases the current flow through the device upon CBZ interaction.

### Amperometric Detection of CBZ

Figure 4B shows the amperometric response of NiSe_2_ sensor upon successive spiking of increase in concentration of CBZ. The experiment was conducted in phosphate-buffered solution of pH 7.2 at a constant voltage of 0.93 V (oxidation potential of CBZ). A constant concentration of 50 nM of CBZ was spiked at a regular interval of 20 s on the device. A quick response was obtained on each addition of CBZ, which was observed because of the oxidation of CBZ at NiSe_2_ surface. This can be clearly attributed to the controlled mass transfer–based chemiresistive sensing of CBZ. The inset of Figure 4B displays the linear calibration graph of the sensor plotted between normalized response vs. concentration of the analyte for n-5 sensors. The obtained regression equation was [I (μA) = 1.99 μM + 4.84] with *R*^2^ = 9,958. The linearity of the device was obtained for CBZ concentration ranging from 50 nM to 10 μM. The sensitivity of the device was obtained as 65.5 μA nM^−1^ cm^−2^. The limit of detection (LOD) was calculated using the formula 3 *S*/*m* (where *S* is standard deviation of the response, and *m* is sensitivity). A low LOD of 18.2 nM is obtained, which is far more superior when compared to the recent reports on CBZ sensing. The excellent response can also be attributed to the large surface area of electroactive NiSe_2_ nanoclusters. The NiSe_2_ provides high conductivity, which shortens the nucleation path enabling easy interaction of CBZ molecules and assists oxidation of CBZ to 2-hydroxy CBZ (Shah et al., 2018) as illustrated in Figure 4C. The Se-rich surface expedites the charge transfer and enhances the catalytic ability. As an additional validity to the electroanalytical performance of NiSe_2_ toward CBZ, DPV studies were also performed. The optimized parameters for DPV studies are given in Supplementary Information Section S2. The CBZ sensing was carried out for two concentrations, namely, a lower concentration (50 nM) and a higher concentration (1 μM) to determine the peak currents as displayed in Figure S2. The anodic peak potential was observed at 0.93 V. The peak current was observed to increase from 7.2 to 23.3 μA for increase in CBZ concentration from 50 nM to 1 μM. Hence, the electrochemical response of the NiSe_2_ sensor was successfully obtained using DPV technique.

### Interference Studies

**Figure 5:**
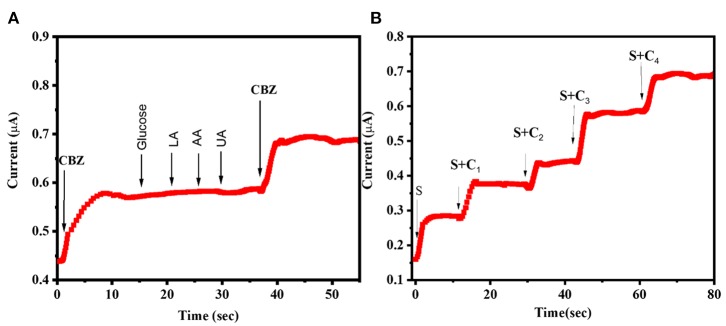
**(A)** Interference studies using amperometric method for successive addition of CBZ (0.1 μM), glucose, lactic acid, ascorbic acid, uric acid, and CBZ (0.1 μM) at an applied potential of 0.93 V. **(B)** Detecting unknown concentrations of CBZ in sweat samples using the fabricated NiSe_2_/PI sensor.

In order to access the suitability of the sensor in detecting CBZ in body fluids, the effect of analytes that coexist with CBZ in sweat samples such as ascorbic acid (AA), glucose, uric acid (UA), and LA on NiSe_2_ surface was investigated. Selectivity studies were performed using amperometric technique as shown in Figure 5A. The results displayed a significant increase in current with CBZ but no/minimal current response obtained for 10-fold concentration of the interfering analytes. The constructed platform displayed excellent selectivity toward CBZ at a constant oxidation potential of 0.93 V. Because the oxidation potentials of the interfering analytes, namely, AA, glucose, UA, and LA are 0.2, 0.25, 0.4, and 0.1 V, respectively (Veeralingam and Badhulika, 2020b), there was no significant response found during the selectivity studies. From the obtained results, it is evident that the NiSe_2_-based sensor displayed excellent selectivity toward CBZ.

### Real-Time Analysis of CBZ in Sweat Samples

The practical utility of the proposed sensor was validated using simulated sweat samples. The real-time sensing and recoverability studies were carried out by initially spiking unknown concentration(s) of CBZ in sweat samples followed by spiking of four known concentrations of CBZ (C1, C2, C3, and C4). The SA method was used to quantify the unknown concentrations of CBZ in the simulated sweat samples. Standard addition method is highly recommended in this case because the sensor displays a linear calibration plot for a wide range of CBZ concentrations. The measured values of current were substituted for each concentration, namely, S, S+ C1, S+ C_2_, S+ C_3_, and S+ C_4_ in the calibration plot as shown in Figure 5B. The obtained linear plot was interpolated such that it cuts the abscissa (X-axis), and the corresponding value represents the unknown concentration of CBZ present in sweat samples (Sha et al., 2019). Table 1 gives the concentrations of CBZ spiked and CBZ recovered in the real-time sweat samples. From the obtained graph, the unknown concentration was quantified as 58 nM. Further, the obtained calibration results map well with the current values, establishing practicability of the 2D-NiSe_2_/PI sensor.

**Table 1:** Determination of unknown concentrations of CBZ in human sweat samples.

**Sample name**	**CBZ spiked**	**CBZ recovered**	**Recovery (%)**
S	–	–	–
S + C_1_	54 nM	53.4 nM	98.8
S + C_2_	98 nM	98.2 nM	100.2
S + C_3_	148 nM	143 nM	96.6
S + C_4_	250 nM	249 nM	100.4

### Stability and Reproducibility Studies

The stability studies of the sensor were performed by measuring the response of the sensor device toward a fixed concentration of CBZ at regular intervals for a duration of 28 days. After every usage, the device was dipped in the buffer solution for a few seconds and dried at 70°C for 30 min and then stored in ideal conditions of 20°C. The response of the sensor is illustrated in Table S1. The relative standard deviation (RSD) was observed to be <0.2%, thus confirming very less from the sensor's response data. The reproducibility of the sensor was investigated by spiking 100 nM concentration of CBZ for *N* = 5 devices. The responses of the sensor recorded are displayed in Table S2. The RSD was observed to be 0.18%. According to the theoretical values, the RSD values <0.2% are considered as a critical criterion for an excellent reproducible sensor (Harazono et al., 2019). The NiSe_2_/PI sensor displayed excellent reproducibility and stability for *N* = 5 different devices. The flexibility of the sensor was evaluated by obtaining the normalized response of the sensor by subjecting it to a strain for 500 bending cycles. The response was measured at regular intervals after bringing back the sensor to normal flat position (unstrained condition) after subjecting it to a strain. The results are shown in Figure S3. A negligible change in response was obtained for 500 bending cycles confirming the excellent robustness. This can be attributed to the strong adhering properties of NiSe_2_ on the PI substrate due to which nanoclusters were not deformed during the bending cycles.

Table 2 summarizes the performance of the as-fabricated NiSe_2_ on PI-based sensor with the previous reports on state-of-the-art 2D materials-based sensors for detection of CBZ. Nanomaterials-based composites such as rGO -SWNT (Unnikrishnan et al., 2012), GO -C_3_N_4_ (Balasubramanian et al., 2018), and graphene-AuNPs (Lavanya et al., 2016) have been used as electrodes for CBZ detection. However, the synthesis procedure involved two steps, namely, (i) Hummers method for graphene-based materials, (ii) chemical synthesis methods such as polymerization and precipitation for SWNT or g-C_3_N_4_. Further, the sensing was carried out using rigid modified GCE electrodes, and some of them involved binders such as Nafion, which reduces the sensitivity of the sensor. The current work involved one-step hydrothermal synthesis of metallic NiSe_2_, and the as-developed NiSe_2_/PI sensor exhibited excellent sensitivity over a wide dynamic linear range of CBZ concentrations. It was further used to accurately determine unknown concentrations of CBZ in real-time sweat samples, and the sensor could be stored and reused up to 28 days. The estimated cost of the sensor was $0.12, which makes it economically viable for healthcare applications. Thus, the developed amperometric platform paves a new path for low-cost, non-enzymatic detection of CBZ in bioanalytes for point-of-care diagnostics.

**Table 2:** Comparison of the NiSe_2_/PI sensor with the previous reports on layered nanomaterials-based sensors.

**Material**	**Synthesis method**	**Detection technique**	**Sensitivity (μA nM^**−1**^)**	LOD* (nM)	**Response time (s)**	**Flexibility**	**References**
Fullerene-C60	Drop dry method	Differential pulse voltammetry	0.59	54	—	No	(Kalanur et al., 2011)
rGO-SWNT	Hummers method	Amperometric	5.12	129	10**	No	(Unnikrishnan et al., 2012)
GO-g-C_3_N_4_	Polymerization	Amperometric	1.73	10.5	24**	No	(Balasubramanian et al., 2018)
Fe-doped SnO_2_	Sol–gel	Square wave voltammetry	0.73	92	—	No	(Lavanya et al., 2016)
Graphene-AuNPs	Deposition-precipitation	Cyclic voltammetry	0.6	303	—	No	(Pruneanu et al., 2011)
NiSe_2_	Hydrothermal method	Amperometric	65.65	18.2	2	Yes	(This work)

* *LOD, limit of detection*.

** *Calculated based on the data given in the reports*.

## Conclusion

To summarize, this work employs a novel yet facile technique for synthesis of high-surface-area metallic 2D-NiSe_2_ nanoclusters and its subsequent use for highly selective and sensitive amperometric detection of CBZ. The synthesized NiSe_2_ was characterized by SEM, XRD, and XPS studies. The sensor exhibits excellent sensitivity of 65.65 μA/nM in the wide linear range of 50 nM to 10 μM CBZ concentrations and a low LOD of 18.2 nM. This enhanced analytical performance can be attributed to the metallic nature of NiSe_2_ and large electroactive surface area of NiSe_2_ nanoclusters. The practical applicability of the sensor was evaluated by measuring its selectivity against interfering analytes such as AA, glucose, UA, and LA and by quantifying unknown concentrations of CBZ in human sweat samples. This strategy presented in this work can be used to develop flexible and wearable sensors for advanced point-of-care medical diagnostics.

## Data Availability Statement

All datasets generated for this study are included in the article/Supplementary Material.

## Author Contributions

SV and SB: conceptualization, methodology, data curation, writing—Original draft preparation, and writing—reviewing and editing.

## Conflict of Interest

The authors declare that the research was conducted in the absence of any commercial or financial relationships that could be construed as a potential conflict of interest.
