# Supplementary material for: Two-Dimensional Metallic NiSe2 Nanoclusters–Based Low-Cost, Flexible, Amperometric Sensor for Detection of Neurological Drug Carbamazepine in Human Sweat Samples
Source: Front Chem. 2020 Apr 30;8:337. doi: 10.3389/fchem.2020.00337 (PMC7205447; doi:10.3389/fchem.2020.00337)
Supplement: Supplementary file 1 [file Data_Sheet_1.docx]

**2D-metallic NiSe_2_ nanoclusters based low cost, flexible, amperometric sensor for detection of neurological drug carbamazepine in human sweat samples**

*Sushmitha Veeralingam^1^ and Sushmee Badhulika^1*^*

*^1^Department of Electrical Engineering, Indian Institute of Technology Hyderabad*

*Hyderabad, 502285, India*

**Corresponding author: E-mail: sbadh@iith.ac.in; Telephone: 040-23018443*

**Section S1. Weight percentage based optimization:**

The PI tape was deposited with 4 different weight percentages of NiSe_2_ namely, 0.1 wt%, 0.2 wt% ,0.3 wt% and 0.4 wt%. Each sensor was spiked with 1 µM of CBZ and the response was calculated using the formulae ΔR/R1= (R1 - R2) / R1, where R1 is the initial resistance of the fabricated NiSe2/PI sensor and R2 being the response of the sensor when exposed to CBZ as shown in figure S2. It was observed that the current increases with increase in NiSe_2_ concentration from 0.1 wt% to 0.2 wt%. With further increase in the NiSe_2_ concentration, the current however decreases. Therefore, current reaches the maximum when concentration (*w*t%) of NiSe_2_ was 0.2% yielding maximum sensitivity to CBZ. The decrease in current with further increase in wt% in response to CBZ can be explained in terms of increase in the thickness of NiSe_2_ deposited which leads to reduction in electrocatalytic active sites available for electro-oxidation of CBZ.

Figure S1. Control sample analysis showing the response of various wt% of NiSe_2_ towards 1 µM of CBZ.

**Section S2: Parameters for DPV studies**

Following are the optimal diﬀerential pulse voltammetry (DPV) parameters used throughout the work: initial potential - 0.5 V; final potential - 1.6 V; increment potential - 0.001 V; amplitude- 0.05 V; pulse width - 0.2 s; pulse period -0.5 s. Before spiking CBZ, a low background current was observed whereas after spiking CBZ to the Phosphate buffer solution of pH 7.2, a distinguished oxidation peak at ∼0.93 V is observed in DPV. Upon increase in the concentration of CBZ, the anodic peak current increases. Here it is noteworthy to mention that the anodic peak current is observed due to the oxidation of CBZ.

Figure S2. DPV studies for a) GCE; NiSe_2_modified GCE in b) 0.1 M Phosphate buffer solution c) 50 nM of CBZ in 0.1 M Phosphate buffer solution d) 10µM of CBZ in 0.1 M Phosphate buffer solution

**Section.S3. Reproducibility and stability of the flexible CBZ sensor:**

Table S3.1. Stability studies of the sensor array.

| **Day** | **Response (∆R/R) of the sensor towards neurological drugs (Conc.-100 nM)** |
| --- | --- |
| Day 1 | 0.58 |
| Day 8 | 0.6 |
| Day 15 | 0.85 |
| Day 28 | 0.9 |
| Relative standard deviation | 0.16 % |

Table S3.2 Reproducibility studies.

| **Device** | **Response (∆R/R) of the sensor towards CBZ (Conc. -100 nM)** |
| --- | --- |
| D1 | 0.53 |
| D2 | 0.46 |
| D3 | 0.63 |
| D4 | 0.84 |
| D5 | 0.89 |
| Relative standard deviation | 0.18 % |

Figure S3. The normalised response obtained for the NiSe_2_/PI sensor when subjected to 500 bending cycles

Figure S4. ln(I) versus (V)^1/2^ plot for the NiSe_2_ /PI device.
